# Efficacy of Selected Live Biotherapeutic Candidates to Inhibit the Interaction of an Adhesive-Invasive *Escherichia coli* Strain with Caco-2, HT29-MTX Cells and Their Co-Culture

**DOI:** 10.3390/biomedicines10092245

**Published:** 2022-09-09

**Authors:** Bronwyn Smit, Chiemeka C. Chinaka, Albert A. Scott, Kirsten Gaiduschek, Eva Hatje, Anna Kuballa, Samantha Coulson, Wayne Finlayson, Mohammad Katouli

**Affiliations:** 1 Centre for Bioinnovation and School of Science, Technology and Engineering University of the Sunshine Coast, Maroochydore 4558, Australia; 2 School of Biomedical Sciences, Faculty of Health, Queensland University of Technology, Brisbane 4000, Australia; 3 School of Health and Behavioural Sciences, University of the Sunshine Coast, Maroochydore 4558, Australia; 4 Servatus Biopharmaceuticals, Coolum Beach 4573, Australia

**Keywords:** AIEC, live biotherapeutic products, Caco-2:HT-29-MTX, adhesion, invasion

## Abstract

Adherent-invasive *Escherichia coli* (AIEC) has been implicated as a microbiological factor in the pathogenesis of inflammatory bowel disease (IBD). We evaluated the ability of six live biotherapeutic products (LBPs) to inhibit the interaction of an AIEC strain to three cell lines representing human gut epithelium. Co-inoculation of LBPs with AIEC showed a reduction in adhesion (up to 73%) and invasion of AIEC (up to 89%). Pre-inoculation of LBPs in HT-29-MTX and Caco-2 cells before challenging with AIEC further reduced the adhesion and invasion of the AIEC, with three LBPs showing significantly (*p* < 0.0001) higher efficiency in reducing the adhesion of AIEC. In co-inoculation experiments, the highest reduction in adhesion (73%) of AIEC was observed in HT-29-MTX cells, whereas the highest reduction in invasion (89%) was seen in HT-29-MTX and the co-culture of cells. Pre-inoculation of LBPs further reduced the invasion of AIEC with highest reduction (97%) observed in co-culture of cells. Our results indicated that whilst there were differences in the efficacy of LBPs, they all reduced interaction of AIEC with cell lines representing gut epithelium. Their efficiency was higher when they were pre-inoculated onto the cells, suggesting their potential as candidates for alleviating pathogenesis of AIEC in patients with IBD.

## 1. Introduction

Inflammatory bowel disease (IBD) is a complex chronic idiopathic condition characterized by chronic bowel inflammation [1]. It consists of two main subtypes: Crohn’s disease (CD) which can occur at any point along the gastrointestinal (GI) tract, and ulcerative colitis (UC), which is limited to the distal colon and rectum [2,3]. Both CD and UC are associated with an increased risk of developing colitis-associated cancer, mainly colorectal cancer (CRC) [4,5,6,7,8]. Although the precise mechanisms underlying IBD pathogenesis are yet to be fully understood, *Escherichia coli* has been implicated as a microbiological factor in disease pathogenesis [9]. Several studies have reported an increase in the relative abundance of mucosa-associated *E. coli* among patients with UC, CD and CRC [10]. Phenotypic characterization of these strains has demonstrated their enhanced ability to diffusely adhere to and colonize intestinal cell surfaces in order to instigate, replicate and drive proinflammatory activities leading to an increase in the severity of ileal inflammatory disease [11]. These bacteria then invade intestinal epithelial cells, and survive and replicate within macrophages. Based on these distinct characteristics, such *E. coli* strains were designated adherent-invasive *E. coli* (AIEC). AIEC also carry several virulence genes not typically found among commensal *E. coli* strains [12].

Lactic acid bacteria and, in particular, lactobacilli can provide significant beneficial roles in supporting the balance of a healthy gut microbiota by maintaining gut barrier integrity, supporting healthy digestive and immune function and suppressing the growth and adhesion of pathobionts [13,14]. Introducing these beneficial microorganisms into the body can be achieved in the form of conventional foods and dietary supplements, such as fermented food and probiotics, or as drugs, which are termed live biotherapeutic products (LBP). The US Food and Drug Administration (FDA) defines LBPs as “biological products that: (1) contain live bacteria; (2) are applicable to the prevention, treatment or cure of a disease or condition of human beings; and (3) are not vaccines” [15]. Introduced beneficial microbes can antagonize pathogens by direct interaction and secretion of antimicrobial substances and/or by interacting with the gut microbiota to produce antimicrobial agents that inhibit the growth of pathogenic species and improve gut barrier protection [16,17]. Evidence of this interaction between *Lactobacillus* strains and the gut epithelium has been shown using T84 cells serving as an epithelial barrier model, where *Lactobacillus* strains modulated the transcription of E-cadherin and β-catenin involved in maintaining adherence junction complexes [18]. Furthermore, lactobacilli have been shown to effectively adhere to both healthy and IBD colonic mucosa and exert local anti-inflammatory effects, both ex vivo and in vivo [19,20]. They further interact with the epithelial cells to promote secretion of glycoproteins, such as mucin and to prevent the adhesion of pathogenic bacteria [21].

It is generally accepted that intestinal microbiota and/or their metabolic products, are likely an important factor involved in the pathogenesis of IBD. It is also accepted that treatment with antimicrobials to alleviate symptoms can also be associated with side-effects, such as bacterial overgrowth [22]. With the widespread concern in progressive antimicrobial resistance and the risk in the potential development of resistant *Candida* spp. and *Clostridioides* (*Clostridium*) *difficile* infections with subsequent disease flares, it is critical to reduce the prescription of broad-spectrum antibiotics when possible [23,24,25]. Furthermore, administration of antibiotics has been shown to enhance AIEC colonization of the gut and mesenteric tissues [12]. This concern raises the need to identify alternatives to antimicrobials, such as LBPs which can compete effectively with pathogens and be used as a prophylactic and/or treatment for IBD without contributing to the rise of antimicrobial resistance.

In view of the above, this study was undertaken to explore the ability of selected *Lactobacillus* strains believed to be novel candidates as LBPs to colonize gut epithelium and to competitively exclude AIEC strains. We hypothesized that such competition will consequently inhibit or reduce the rate of AIEC colonization of, and invasion into, the intestinal epithelial cells and, therefore, they can be used as either a prophylactic or treatment option to prevent or alleviate symptoms associated with IBD. However, most studies assessing the interaction of LBP candidates utilize only one epithelial cell line which may not completely mimic the characteristics of the gut epithelium. Here we validated the efficacy of our LBP candidates to inhibit or reduce interaction of the AIEC strain F44A-1 with two cell lines i.e., Caco-2 and HT-29-MTX cells. These cell lines are derived from human colon adenocarcinoma and have been successfully used in many in vitro studies. Caco-2 cells can be differentiated in the culture medium to form a polarized cell monolayer with tight junctions and microvilli to resemble important characteristics of human intestinal mature enterocytes. The main drawback of this cell line is that it does not produce a mucus layer. The other cell line, HT-29, with methotrexate (MTX) adaptation i.e., HT-29-MTX cell line, differentiates in culture media to secret mucin [26,27,28]. Mucus-secreting HT-29-MTX subclones have previously been isolated and characterized regarding tight junction formation, development of confluent monolayers and production of mucin [29]. On the other hand, co-culture of Caco-2 and HT-29-MTX cells has been postulated to reflect the cellular components of the intestine more effectively than the monoculture [30]. Therefore, we extended our investigation by including the co-culture of Caco-2 and HT-29-MTX cells to compare the efficacy of our selected LBPs to inhibit adhesion and invasion of a well characterized AIEC strain.

## 2. Materials and Methods

### 2.1. Bacterial Strains and Culture Conditions

The AIEC strain F44A-1 was previously isolated from a patient with colorectal cancer and carried all phenotypic and genotypic characteristics consistent with AIEC strains including the presence of AIEC-associated virulence genes, their diffuse adhesion pattern to Caco-2 cells as well as survival and replication in macrophages [31]. The low-adhering and non-invasive *E. coli* 46-4 was sourced from animals subjected to starvation and hemorrhage [32] and served as a negative control strain. LBP candidates used in this study were supplied by Servatus Biopharmaceuticals and included *Lactobacillus* strains SVT 01D1, SVT 04P1, SVT 05P2, SVT 06B1, SVT 07R1 and SVT 08Z1. The AIEC strain was maintained at −80 °C in Luria-Bertani (LB) broth (Merck, Rahway, NJ, USA) with 20% glycerol. It was streaked on MacConkey agar first to check for its purity before growing on nutrient agar as a working culture and regrown in LB broth in a reciprocal shaker (140 strokes min^−1^) at 37 °C for 24 h before each adhesion or invasion assay. All LBP strains were maintained at −80 °C in de Man, Rogosa, Sharpe (MRS) broth (Oxoid, Scoresby, Victoria, Australia) with 20% glycerol. They were grown on MRS agar as a working culture and regrown in MRS broth for 48 h at 37 °C before each assay.

### 2.2. Cell Lines and Cell Culture

Two human colon adenocarcinoma cells, Caco-2 cells (ATCC^®^ HTB-37) alone, and HT-29-MTX-E12 cells (ECACC 12040401), originally differentiated from the HT-29 (ATCC^®^ HTB-38) cell into mucin producing mature goblet cells using methotrexate (MTX) [33], as well as a co-culture of both cell lines. Caco-2 cells in culture medium forms a polarized cell monolayer with tight junctions and microvilli to resemble important characteristics of human intestinal mature enterocytes. Cells were grown in 50 mL culture flasks (Grenier Bio-one, Australia) to confluence in Eagle’s Minimum Essential Medium (EMEM, Sigma Aldrich, St. Louis, MO, USA) and supplemented with 20% (*v*/*v*) fetal bovine serum (FBS) (Lonza, Brisbane, Australia) for Caco-2 cells and 15% (*v*/*v*) FBS for HT29-MTX cells and 1% (*v*/*v*) penicillin-streptomycin (Thermo Fisher, Brisbane, Australia). Co-culture of Caco-2 and HT-29-MTX cell lines was prepared by seeding Caco-2 and HT-29-MTX cells at a ratio of 9:1 and grown to confluence. The monolayer formed in this way could secrete a sufficient amount of mucin whilst expressing tight junctions and microvilli brush borders similar to that of the GI epithelium [34].

All cell cultures were maintained at 37 °C in an atmosphere of 5% CO_2_. Culture media was changed every 48 h. At confluence, cells were sub-cultured into the eight-well chamber slides (Nunc Lab-Tek II) for adhesion assays and into sterile 96-well flat bottom plates for invasion assays.

### 2.3. Adhesion Assays

The adhesion of AIEC alone, LBPs strains alone, and AIEC in the presence of the LBP strains was tested on both cell lines and their co-culture using methods described previously [35], with modifications from previous protocols [36,37]. Before the adhesion assay, cell lines were seeded onto an eight-well glass chamber slide system (Nunc Lab-Tek II). The cells were grown to ~75% confluence and, prior to the assay, the medium was replaced with antibiotic-free medium. The AIEC strain was cultured in LB broth (Merck) overnight at 37 °C with agitation (140 strokes. min^−1^) and LBP strains were cultured in MRS broth (Oxoid, Scoresby, Victoria, Australia) for 18 h at 37 °C. Bacterial suspensions were centrifuged at 3500 rpm for 12 min and the supernatant was discarded. The pellets were resuspended in phosphate-buffered saline (PBS) (pH 7.4), and 100 μL of the original suspension (10^9^ colony-forming units, CFU. mL^−1^, OD = 1 at 600 nm) was inoculated into the appropriate chambers to give a multiplicity of infection (MOI) of 100 after counting the number of cells in three wells and adjusting the concentration of bacterial suspension.

For the competitive adhesion, LBP strains and the AIEC were co-inoculated at an identical concentration, i.e., 10^9^ CFU.mL^−1^ prior to incubation at 37 °C for 90 min. To assess the ability of LBP strains to prophylactically inhibit adhesion of AIEC, 100 µL of each LBP suspension (10^9^ CFU mL^−1^) were pre-inoculated for 60 min onto the wells before infecting cells with the same concentration of AIEC. Cells were then incubated at 37 °C for a further 90 min and non-adherent bacteria were removed by washing the wells three times with PBS (pH 7.4). Cells were then fixed with 95% ethanol (*v*/*v*) for 5 min and stained using Gram staining to differentiate between Gram-positive LBPs (*Lactobacillus* strains) and Gram-negative AIEC and their adhesion was observed under a light microscope. The extent of colonization of the gut epithelium by both AIEC and LBP strains was determined by randomly selecting 100 cells and counting how many showed bacterial adhesion, while the ability of the strains to interact with each cell was determined by counting the bacterial number on 25 randomly selected cells showing adhesion.

### 2.4. Invasion Assay

For the invasion assay, a similar procedure was used as before [38] with some modifications. Caco-2, HT-29-MTX and a co-culture of both cells (9:1 ratio) were grown onto a flat bottom 96-well plate until full confluence in EMEM medium similar to the adhesion assay. LBP strains and AIEC were also grown as described in the adhesion assay and inoculated onto Caco-2, HT-29-MTX and their co-culture in the same manner as described for adhesion to yield a MOI of 100. For competitive invasion, LBP strains and AIEC were co-inoculated followed by a 2 h incubation for Caco-2 and HT-29-MTX cells and 90 min incubation for the co-culture at 37 °C. To assess the ability of LBP strains to prophylactically inhibit or decrease AIEC invasion, they were pre-inoculated onto the cells and incubated for 60 min prior to infection with the same concentration of AIEC, and the wells were incubated at 37 °C for 2 h for Caco-2 and HT-29-MTX and 90 min for co-culture. Wells were then inoculated with gentamicin (150µL mL^−1^) (Gibco, Victoria, Australia) for 60 min to kill any extracellular bacteria and the contents were removed and washed three times with PBS and lysed with 0.1% (*v*/*v*) Triton X-100 (Sigma-Aldrich) to release the invading AIEC. The lysate was then serially diluted and 100 μL volumes were plated onto MacConkey Agar No. 3 (Oxoid, Scoresby, Victoria, Australia) plates and incubated for 24 h at 37 °C before colonies were counted. Mean ± SEM number of bacteria were calculated with due corrections for the dilution factors. *E. coli* strain 46-4 was used as a negative control for both assays at the same concentration of AIEC.

### 2.5. Statistical Analysis

All adhesion and invasion experiments were carried out in triplicates. GraphPad Prism statistical software (Version 8.0.0) was used for statistical analysis. Two-way ANOVA followed by Tukey’s multiple comparisons test were used to determine the differences in the mean level of adhesion and invasion among strains across all test groups, and the unpaired t test was used for comparing the difference between co-inoculation and pre-inoculation of each LBP. Correlation between adhesion and invasion capabilities of strains were evaluated using Pearson’s correlation coefficient. Differences were considered statistically significant if *p* < 0.05.

## 3. Results

### 3.1. Colonization Experiment

**Figure 1:**
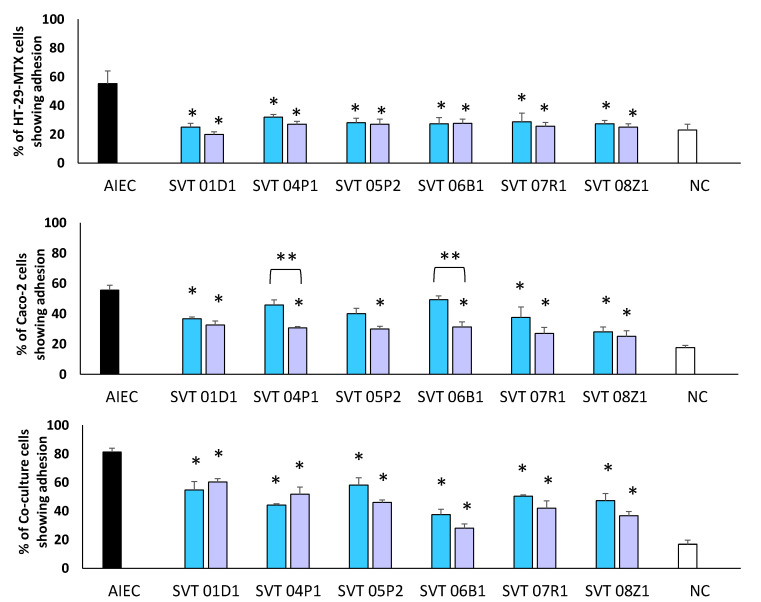
Colonization of HT-29-MTX, Caco-2 and co-culture of Caco-2/HT-29-MTX cells by AIEC alone 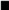, AIEC when co-inoculated with the LBPs 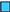, AIEC in the presence of pre-inoculated LBPs 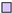 and negative control [NC] 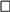. * Significant differences between the percentages of cells showing adhesion with AIEC before addition of LBPs strains in co-inoculation or pre-inoculation experiments; *p* ≤ 0.0001 (HT-29-MTX), *p* ≤ 0.0001 (Caco-2), *p* ≤ 0.0001 (Co-culture). ** Significant difference between co-inoculation and pre-inoculation for SVT 06B1 (*p* = 0.03) in Caco-2.

When inoculated alone, AIEC colonized 55% of the Caco-2 and HT-29-MTX cells and more than 81% of the co-culture cells (Figure 1). In both co-inoculation and pre-inoculation experiments, there was a significant (*p* < 0.0001) reduction in AIEC colonization of monoculture and co-culture cells, with a generally higher reduction in pre-inoculation experiments (Figure 1). Whilst all six LBPs reduced colonization in the HT-29-MTX cell monoculture, only three LBP strains, i.e., SVT 01D1 (*p* = 0.015), SVT 07R1 (*p* = 0.027) and SVT 08Z1 (*p* < 0.0001) significantly reduced the colonization of AIEC in the Caco-2 cells following co-inoculation (Figure 1). In the co-culture of both cell lines a significant reduction in colonization was seen with all LBPs (*p* < 0.0001) (Figure 1). Pre-inoculation of LBP strains further reduced colonization of the AIEC in HT-29-MTX and Caco-2 cell lines with only SVT 04P1 and SVT 06B1 showing a significant difference (*p* = 0.01 for both) between the co-inoculation and pre-inoculation experiments (Figure 1).

**Figure 2:**
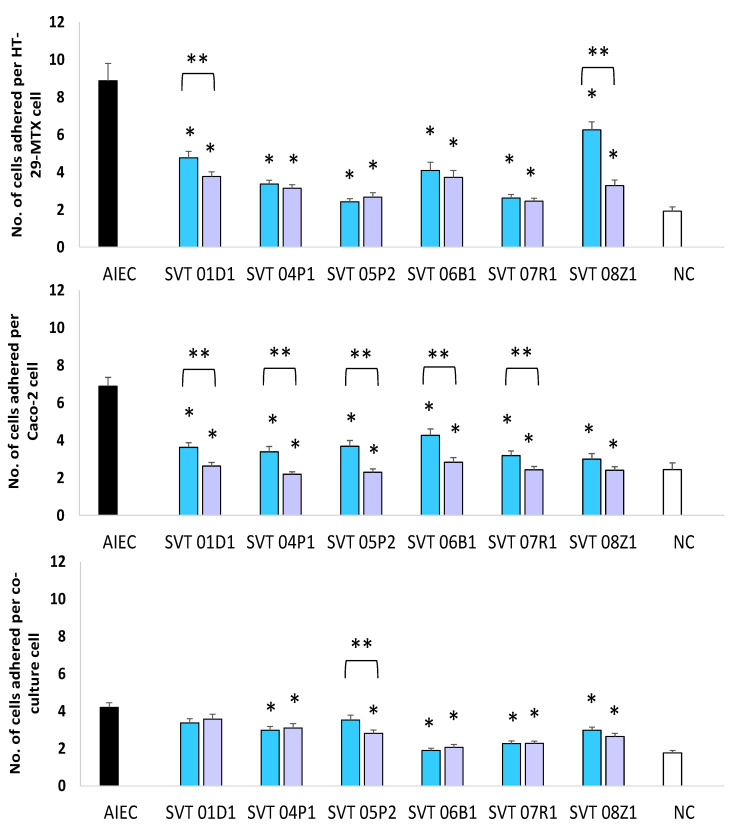
The number of AIEC cells adhering per cell on HT-29-MTX, Caco-2 and co-culture of Caco-2/HT-29-MTX cells. AIEC alone 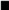, AIEC when co-inoculated with the LBPs 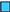, AIEC when cells were pre-inoculated with LBPs 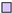 and negative control [NC] 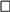. Significant differences (*) were observed before and after the addition of LBP strains (co-inoculation or pre-inoculation); *p* < 0.0001 (HT-29-MTX), *p* < 0.0001 (Caco-2), *p* < 0.0001 (Co-culture). ** Significant difference between co-inoculation and pre-inoculation for SVT 05P2 (*p* = 0.049) and SVT 06B1 (*p* = 0.04) in Caco-2, and SVT 08Z1 (*p* < 0.0001) in HT-29-MTX.

The number of AIEC strains adhering to individual cells of all three cell cultures was also reduced significantly (*p* < 0.0001) in the presence of most LBPs in both co-inoculation and pre-inoculation experiments (Figure 2). The percentage reduction of AIEC adhesion by LBP strains in both the co-and pre-inoculation experiments has been summarized in Table 1. Overall, pre-inoculation resulted in a greater reduction in adhesion of AIEC compared to co-inoculation (Table 1). AIEC on its own showed a significantly better ability to adhere to HT29-MTX cells (8.9 ± 0.9) (*p* < 0.0001) and Caco-2 cells (6.9 ± 0.5) (*p* = 0.0066) than to the co-culture model (4.2 ± 0.2) (Figure 2).

### 3.2. Invasion of AIEC

**Figure 3:**
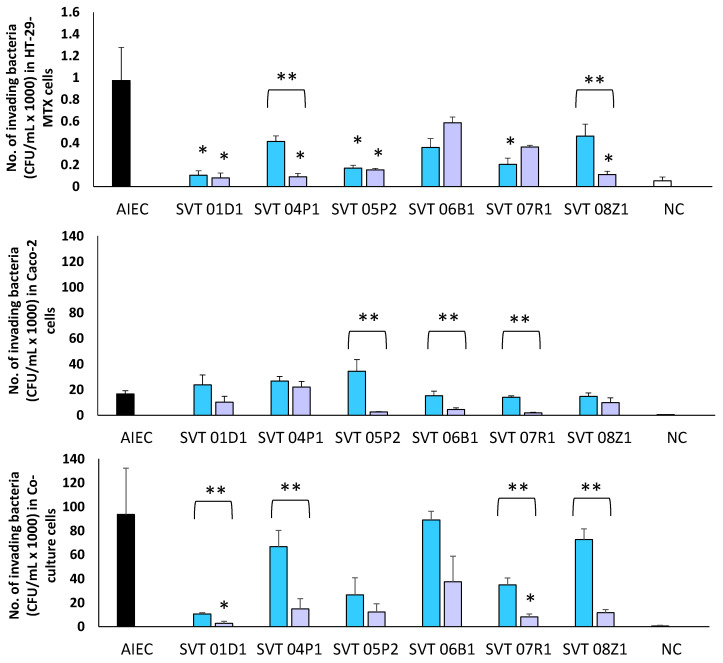
The number of cells showing invasion of AIEC on HT-29-MTX, Caco-2 and co-culture cells. Invading AIEC alone 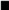, AIEC when co-inoculated with the LBPs 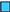, AIEC in the presence of pre-inoculated LPBs 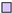 and negative control [NC] 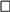. *p* < 0.0001 (HT-29-MTX), * *p* < 0.0001 (Caco-2), * *p* < 0.0001 (Co-culture). ** Significant difference between co-inoculation and pre-inoculation for SVT 05P2 (*p* = 0.0003) in Caco-2.

The ability of LBPs to reduce invasion of the AIEC was varied among cell lines. In Caco-2 cells, there was no significant reduction in the number of invading AIEC when treated with LBP strains for either pre- or co-inoculation (Figure 3). HT-29-MTX cells on the other hand, were more efficient in reducing the invasion of AIEC than in Caco-2 cells although this ability was strain dependent (Figure 3). The percentage reduction of AIEC invasion by LBP strains in both the co-and pre-inoculation experiments has been summarized in Table 2. Again, in all experiments, pre-inoculation of LBPs mostly resulted in higher reduction of invasion with some LBP strains almost completely inhibiting the invasion of AIEC for example, in the co-culture assay SVT 01D1 (97%, *p* = 0.02) and SVT 07R1 (97%, *p* = 0.04) both significantly impaired invasion. All the LBPs showed a reduction following pre-inoculation when compared with co-inoculation results, however this again was cell-line dependent (Table 2).

### 3.3. Correlation between Adhesion and Invasion

In HT29-MTX cells, there was a positive correlation (0.46) between the number of AIEC adhering per cell and the number invading in the co-inoculation experiment but not in pre-inoculation (0.07) (Table 3). This, however, was not seen in the Caco-2 or co-culture cell models. A higher number of LBPs adhering to HT-29-MTX cells, correlated with a higher reduction in adhesion (0.26 for co-inoculation; 0.50 for pre-inoculation) and invasion of AIEC (0.34 for pre-inoculation). In Caco-2, a reduction in AIEC adhesion was positively correlated with higher numbers of adhering LBP cells in both co-inoculation (0.33) and pre-inoculation (0.27). Similarly, LBP adherence was positively correlated (0.36) to a reduction in AIEC invasion following pre-inoculation in the co-culture. Interestingly, the co-culture showed no correlation between LBP adhesion and AIEC adhesion in either co-inoculation (−0.01) or pre-inoculation (−0.13). In most cases, the Pearson coefficients were low to moderate, and the only value of statistical significance was seen between adhesion of LBPs and the reduction of AIEC in the pre-inoculation experiment.

## 4. Discussion

The etiology of IBD, whilst not completely understood, is multifactorial in nature involving genetic susceptibility, external environmental factors, defective immune responses and alterations in the intestinal microbial composition [2,39,40]. A gastrointestinal dysbiotic state has been established in that there is a significant reduction in microbial diversity in IBD patients compared to healthy controls [39,41] and often predominated by pathobiont microbes [2,40]. This trend has been observed in patients with CD who exhibit a higher abundance and richness of pathogens such as AIEC pathovar in their biopsy samples [42,43], with a marked increase relative to the severity of bowel inflammation [44]. This raises the need to ameliorate the impact of these pathogenic bacteria by reducing their interaction with the gut epithelium using LBPs that could be beneficial for the treatment and maintenance of IBD remission.

The mechanism of action of the LBPs may include the ability to effectively adhere to the intestinal mucosa and inhibit/reduce the ability of AIEC strains to adhere and colonize the gut epithelium, providing clinically meaningful outcomes. In this study, we demonstrated that the selected LBP *Lactobacillus* candidates reduced the adherence of AIEC to two cell lines that represent the intestinal epithelium, as well as their co-culture. This is supported by previous studies where *Lactobacillus* strains were shown to inhibit cell association and adhesion of some pathogens such as enterovirulant *E. coli*, *Enterococcus faecalis* and *Salmonella typhimurium* to Caco-2 cells [45,46].

The AIEC strain used in this study was shown to be a highly adherent and invasive strain in our previous study via the presence of virulence genes typically found among AIEC strains, as well as its ability to survive and replicate within macrophages [46]. This AIEC strain colonized more than 55% of the cells of Caco-2 and HT-29-MTX cultures and even higher in the co-culture of both cell lines. Caco-2 cells have been used in many studies to assess the adhesive ability of bacterial strains [30,47,48,49]. This cell line produces microvilli and tight junctions, although they normally do not produce mucus. In contrast, HT-29 cells are differentiated into mature goblet cells using methotrexate (MTX) to form HT-29-MTX. We have previously used the co-culture of Caco-2 and HT-29-MTX to assess the ability of adhesion of AIEC strains isolated from different sources and have shown that AIEC strains bind to this co-culture efficiently with a typical diffuse adhesion pattern [31].

In testing the ability of LBPs against AIEC, we used two methods of assessment. In co-inoculation studies, we aimed to evaluate the ability of LBPs to competitively exclude the pathogen-a mechanism that may lead to treatment of the IBD. We also assessed the ability of the LBPs to prophylactically inhibit colonization of the pathogen by pre-incubating cell lines for a period of 60 min to allow for an initial colonization of the LBP before challenging them with the pathogen. Time dependent inhibition of pathogen adhesion is a key trait, especially if they are to be used as prophylactics. This has been shown previously where pre-inoculation with *L. casei* resulted in a decrease in AIEC LF82 adhesion to intestinal cells [50]. In the present study, pre-inoculation of the cells with LBPs, although further reduced adhesion of the AIEC, did not completely inhibit colonization of this pathogen. We also found a positive correlation between the number of LBP cells adhering to the cell lines and a reduction in adhesion and invasion of AIEC; however, this was dependent on cell line and experimental conditions (i.e., co-inoculation versus pre-inoculation). These findings collectively suggest that the LBP strains used in this study can be considered as good candidates for both competitive exclusions, i.e., treatment, and as prophylactic measures in patients where IBD is associated with AIEC. Further studies using longer pre-inoculation periods would be necessary to determine the true efficacy of the LBPs in completely excluding AIEC strains from adhesion to host cells.

To be of clinical benefit to the host, LBPs are expected to modulate intestinal pathobiont abundance and activity and protect the GI tract from environmental pathogens. Production of mucin by goblet cells in the GI tract is important to replenish and maintain the mucus barrier; however, this function can be disrupted by bacteria and their toxins. This can lead to pathological conditions like chronic inflammatory diseases [51]. In co-inoculation and pre-inoculation experiments many of the LBP strains were able to significantly reduce the invasion of the AIEC strain F44A-1 in cell lines that were covered with mucin (i.e., HT-29-MTX and co-culture of Caco-2:HT-29-MTX cells) as opposed to Caco-2 cells alone that are void of mucin. This may suggest that production of mucin is an important characteristic in these cell lines against adhesion or invasion of AIEC strains. It must be noted, however, that some lactobacillus strains are able to regulate mucin production through up regulating mucin gene expression [51]. In our study, we did not see a higher invasion of AIEC in non-mucin producing Caco-2 cells which could be partly due to the decreased paracellular permeability in this cell line as shown previously [52]. Here, in addition to competitive binding, another aspect to consider is that pre-inoculation may be promoting mucin production in these cell lines. Longer incubation periods and measurement of mucin production may provide further mode of action. Moreover, research to explore suitable concentrations of LBP candidates in an improved model of cells that better mimic the GI tract physiology could shed more light on the suitability of these candidates as biotherapeutic agents for human use.

In conclusion, we showed that the LBP candidates tested in this study significantly reduce the adhesion and invasion of the AIEC F44-1 strain in cell lines used although this efficacy varied among the strains. Pre-inoculation of the cell lines with the LBP candidates followed by the challenge with this pathogen was in most cases associated with a further reduction in adhesion and invasion of AIEC, although in some cases, this was cell line dependent. Further tests are needed to evaluate the potential of these strains before they can be introduced as an option for treatment of IBD.

## Figures and Tables

**Table 1:** The percentage of reduction in adhesion to the cell lines by AIEC and the number of AIEC cells adhering per cell (Mean ± SEM) in the presence of LBP strains (shown in bold letters) in both co-inoculation and pre-inoculation experiments. * Shows significance of reduction in the number of AIEC adhering per cell in the presence of the LBP strain versus the number of AIEC alone. ** Shows significance of reduction in the number of AIEC adhering per cell in pre-inoculation experiments versus co-inoculation. *p*-values that are only significant have been shown.

LBP Strains	HT29-MTX			Caco-2			Caco-2:HT29-MTX		
	Co-Inoculation (No. of Bacteria)	Pre-Inoculation (No. of Bacteria)	*p*-Value Co- vs. Pre-Inoculation	Co-Inoculation (No. of Bacteria)	Pre-Inoculation (No. of Bacteria)	*p*-Value Co- vs. Pre-Inoculation	Co-Inoculation (No. of Bacteria)	Pre-Inoculation (No. of Bacteria)	*p*-Value Co- vs. Pre-Inoculation
**SVT 01D1**	46% (5 ± 0.4) <0.0001 *	57% (4 ± 0.2) <0.0001 *	0.02 **	47% (4 ± 0.3) <0.0001 *	62% (3 ± 0.2) <0.0001 *	0.002 **	20% (3 ± 0.2)	15% (4 ± 0.3)	
**SVT 04P1**	62% (3 ± 0.2) <0.0001 *	65% (3 ± 0.2) <0.0001 *		51% (3 ± 0.3) <0.0001 *	68% (2 ± 0.1) <0.0001 *	0.0001 **	29% (3 ± 0.2) 0.001 *	26% (3 ± 0.2) 0.007 *	
**SVT 05P2**	73% (2 ± 0.2) <0.0001 *	70% (3 ± 0.2) <0.0001 *		46% (4 ± 0.3) <0.0001 *	67% (2 ± 0.2) <0.0001 *	0.0001 **	16% (4 ± 0.3)	33% (3 ± 0.2) <0.0001 *	0.03 **
**SVT 06B1**	54% (4 ± 0.4) <0.0001 *	58% (4 ± 0.4) <0.0001 *		38% (4 ± 0.3) <0.0001 *	59% (3 ± 0.2) <0.0001 *	0.001 **	55% (2 ± 0.1) <0.0001 *	51% (2 ± 0.2) <0.0001 *	
**SVT 07R1**	70% (3 ± 0.2) <0.0001 *	72% (3 ± 0.2) <0.0001 *		54% (3 ± 0.3) <0.0001 *	65% (2 ± 0.2) <0.0001 *	0.02 **	46% (2 ± 0.1) <0.0001 *	46% (2 ± 0.1) <0.0001 *	
**SVT 08Z1**	30% (6 ± 0.4) 0.001 *	63% (3 ± 0.3) <0.0001 *	<0.0001 **	57% (3 ± 0.3) <0.0001 *	65% (2 ± 0.2) <0.0001 *		29% (3 ± 0.2) 0.001 *	37% (3 ± 0.2) <0.0001 *	

**Table 2:** The percentage of reduction in invasion of cell lines by AIEC and the number of AIEC cells invading cell lines (Mean ± SEM) in the presence of LBP strains (shown in bold letters) in both co-inoculation and pre-inoculation experiments. * Shows significance of reduction in the number of AIEC invading cell lines in the presence of the LBP strain versus the number of AIEC alone. ** Shows significance of reduction in the number of AIEC invading cell lines in pre-inoculation experiments versus co-inoculation. *p*-values that are only significant have been shown.

LBP Strains	HT-29-MTX			Caco-2			Caco-2:HT-29-MTX		
	Co-Inoculation (No. of Bacteria)	Pre-Inoculation (No. of Bacteria)	*p*-Value Co- vs. Pre-Inoculation	Co-Inoculation (No. of Bacteria)	Pre-Inoculation (No. of Bacteria)	*p*-Value Co- vs. Pre-Inoculation	Co-Inoculation (No. of Bacteria)	Pre-Inoculation (No. of Bacteria)	*p*-Value Co- vs. Pre-Inoculation
**SVT 01D1**	89% (103 ± 42) 0.001 *	92% (80 ± 46) 0.0008 *		−43% (23667 ± 7881)	38% (10333 ± 4667)		89% (10506 ± 500)	97% (2814 ± 500) 0.02 *	0.02 **
**SVT 04P1**	58% (413 ± 52)	91% (90 ± 30) 0.001 *	0.006 **	−61% (26667 ± 3757)	−33% (22000 ± 4509)		29% (66786 ± 2000)	84% (14914 ± 500)	0.03 **
**SVT 05P2**	83% (170 ± 25) 0.004 *	84% (153 ± 13) 0.003 *		−108% (34333 ± 9262)	85% (2533 ± 371)	0.03 **	72% (26733 ± 1900)	87% (12194 ± 400)	
**SVT 06B1**	63% (360 ± 81)	40% (587 ± 52)		7% (15333 ± 3528)	73% (4467 ± 1369)	0.045 **	5% (89110 ± 600)	60% (37520 ± 200)	
**SVT 07R1**	79% (203 ± 58) 0.006 *	63% (365 ± 15)		15% (14000 ± 1155)	89% (1833 ± 518)	0.0007 **	63% (34894 ± 400)	97% (2626 ± 300) 0.04 *	0.01 **
**SVT 08Z1**	52% (463 ± 110)	89% (110 ± 31) 0.001 *	0.04 **	11% (14667 ± 2848)	41% (9767 ± 3930)		22% (72789 ± 700)	87% (11819 ± 500)	0.003 **

**Table 3:** The correlation between adhesion and invasion of AIEC in HT-29-MTX, Caco-2 and co-culture cells, as well as the correlation between LBP adhesion on the cell lines and reduced adhesion and invasion of AIEC. * Shows significant correlation. *p*-values that are only significant have been shown.

Correlation	HT-29-MTX		Caco-2		Caco-2/HT-29-MTX	
	Co-Inoculation	Pre-Inoculation	Co-Inoculation	Pre-Inoculation	Co-Inoculation	Pre-Inoculation
Between adhesion and invasion of AIEC	0.46	0.07	0.18	−0.42	−0.61	−0.67
Between adhesion of LBPs and reduction in AIEC adhesion	0.26	0.50	0.33	0.27	−0.01	−0.13
Between adhesion of LBPs and reduction in AIEC invasion	−0.29	0.34	−0.01	−0.86 (0.015 *)	−0.04	0.36

## Data Availability

Data available from corresponding author upon reasonable request.
